# Then Tell Me What You Think About Morality: A Freedom of Expression Perspective on the CJEU’s Decision in *FACK JU GÖHTE* (C-240/18 P)

**DOI:** 10.1007/s40319-020-00936-9

**Published:** 2020-04-28

**Authors:** Tobias Endrich-Laimböck, Svenja Schenk

**Affiliations:** 1 MJur (Oxon); Doctoral Student, Ludwig-Maximilians-Universität, Munich, Germany; 2 Doctoral Student, Goethe-University, Frankfurt am Main, Germany

**Keywords:** Morality bars, Freedom of expression, Principles of morality, *Fack Ju Göhte*

## Abstract

This case note on the CJEU’s decision in *Fack Ju Göhte* focuses on morality bars and freedom of expression in EU trade mark law. Based on the presumption that a refusal to register a trade mark for being contrary to accepted principles of morality interferes with freedom of expression, an empirical concept of morality appears incompatible with freedom of expression. However, the test for morality developed by the CJEU seems to employ such an empirical concept at least partially. In addition, the test seems to lack legal certainty. In light of freedom of expression, both represent a potential shortcoming of the decision.

## Introduction

The judgment in C-240/18 P – *Fack Ju Göhte*, handed down by the Court of Justice of the European Union (CJEU) on 27 February 2020, concerned the interpretation of the absolute ground of refusal for marks being contrary to accepted principles of morality under Art. 7(1)(f) of Regulation (EU) 2017/1001 of the European Parliament and of the Council of 14 June 2017 on the European Union trade mark^1^ (EUTMR). This was the first time the CJEU has given a judgment on this provision. Starting from the presumption that Art. 7(1)(f) EUTMR interferes with freedom of expression (2), we argue that applying an empirical test under Art. 7(1)(f) EUTMR is incompatible with freedom of expression (3). We then assess whether and to what extent the test developed by the CJEU is empirical (4), before pointing out potential shortcomings of that test (5).

## Art. 7(1)(f) EUTMR Interferes with Freedom of Expression

While the General Court (GC) at first instance stated that “in the field of art, culture and literature, there is a constant striving to preserve freedom of expression, which does not exist in the field of trade mark law”,^2^ the Advocate General (AG) disagreed with this statement,^3^ as did the CJEU.^4^ The CJEU’s finding that “freedom of expression, enshrined in Article 11 of the Charter of Fundamental Rights of the European Union, must […] be taken into account when applying Article 7(1)(f)” is in line with its settled case law according to which all measures of the EU must observe fundamental rights^5^ and was also prompted by the recitals of the EUTMR.^6^ Although the GC’s statement was corrected, the judgment remains silent on how freedom of expression should have affected the outcome of the decision. Freedom of expression was not considered in the CJEU’s interpretation of Art. 7(1)(f) EUTMR. The AG also explicitly wondered what the confirmation that freedom of expression applies in the field of trade mark law “brings to the solution of the present case”,^7^ without further pursuing this route. He pointed out that “the Appellant also had some difficulty in explaining precisely how expressly taking on board freedom of expression should have altered the test under Article 7(1)(f)”.^8^

However, if the refusal to register a mark interferes with freedom of expression as enshrined in both Art. 11 of the Charter of Fundamental Rights of the EU (CFR) and Art. 10 of the European Convention on Human Rights (ECHR), this makes for an argument regarding the interpretation of Art. 7(1)(f) EUTMR. The provision would have to be interpreted in a way which makes this inference justified. The interference with freedom of expression and/or a serious issue with its justification in relation to morality bars have generally been contested by the EUIPO as well as (national) courts and legal scholars.^9^ However, according to the European Court of Human Rights (ECtHR), the refusal of a trade mark application amounts to an interference with the applicant's exercise of their freedom of expression and requires justification.^10^

## Incompatibility of an Empirical Approach to Morality with Freedom of Expression

If an empirical approach to morality under Art. 7(1)(f) EUTMR was incompatible with freedom of expression, this justificatory requirement would then preclude an interpretation resulting in the application of an empirical approach to morality.

### Conceptual Distinction: Empirical versus Normative Approach to Morality

An empirical approach treats morality as a matter of fact instead of reasoning. The individual subjective beliefs (values) of the members of society form the benchmark, not because these values are by themselves objectively binding norms, but because they are the empirical fact that this approach deems decisive.^11^ Thus, the empirical approach can be characterised as descriptive, inductive or “bottom-up”.^12^ It can be contrasted with a normative (or “top-down”) approach under which objective values are deduced from normative sources, e.g. positive law.^13^ Under such normative approach, these values are, as such, binding. Morality, then, is a matter of reasoning, not of facts. Subjective values and objective values may match, but it is the particular approach that matters, not the individual outcome of a case. It is not the “correct” result – immoral or not – that justifies an interference with freedom of expression. It is the result, i.e. the interference, that must be justified. Also, it does not follow from our argument that a normative/top-down approach is justified. To stay clear of any false-alternative argument, additional questions would have to be answered to make that claim, e.g. whether binding, objective values can be found outside of positive law.

### Justifying Interference: Necessary in a Democratic Society

Under Art. 10(2) ECHR, an interference with freedom of expression is only justified if it is prescribed by law, pursues a legitimate aim and is necessary in a democratic society.^14^ It is the requirement of necessity where an empirical approach fails, because it would most likely render the interference of Art. 7(1)(f) EUTMR with freedom of speech unproportionate (3.3), while it is also incompatible with the underlying values of the ECHR (3.4).

### An Empirical Approach is Unproportionate

To be necessary in a democratic society under Art. 10(2) ECHR, the interfering measure must be proportionate to the legitimate aim pursued.^15^ An analysis of Art. 7(1)(f) EUTMR could thus start by identifying its aim, then find out whether it is legitimate according to the list in Art. 10(2) ECHR^16^ and whether the provision is proportionate to this aim. Applying this requirement to the refusal of a trademark application, the ECtHR found a wide margin of appreciation for the regulation of advertising when assessing its proportionality.^17^

The CJEU’s judgment remains silent on the rationale behind Art. 7(1)(f) EUTMR. We will therefore assess the proportionality of an empirical approach to morality on a hypothetical level, that is in relation to the aims which are most often being proposed as standing behind morality bars to trade mark registration,^18^ namely to support objective values in society (3.3.1), to reduce harm (3.3.2), and to prevent the perception of endorsement (3.3.3). Also, we will discuss the proposal to treat morality as a subcategory of distinctiveness (3.3.4).

#### Guiding Society by Supporting Objective Values

Regarding the idea that morality bars serve as a policy tool to guide society towards specific objective moral values,^19^ it suffices to point to the aforementioned detachment of subjective moral beliefs as currently present in society from the objective moral values which are to be introduced. For that reason, an empirical approach does not assist the state’s attempts to enforce objective moral values.^20^ Instead, it turns the *is* into an *ought*, merely preserving the status quo of individual beliefs, independent of an objective framework of moral values. Additionally, the refusal to register cannot even effectively preserve the status quo, since the individual beliefs are due to change for reasons other than (non-)registration of a trade mark, because the refusal to register a sign in itself does not hinder (even commercial) usage of such sign due to the nature of a trade mark as a negative right. All that an empirical approach appears to do is slow down societal change without any apparent justification for such preservation. If one wants to see Art. 7(1)(f) EUTMR as a policy tool to strengthen objective values, its efficacy under an empirical approach is a matter of coincidence, that is whether the supported objective values match the status quo of subjective beliefs.

#### Reducing Harm (Negative Emotions)

An empirical approach also faces problems in explaining that Art. 7(1)(f) EUTMR is effective in reducing negative effects caused by “immoral” trade marks. Assuming that Art. 7(1)(f) EUTMR aims to raise public welfare and health by reducing the number of instances where people encounter trade marks which are contrary to their personal beliefs and thus to reduce negative emotions caused by such encounters,^21^ this appears to favor *prima facie* an empirical approach. Indeed, an empirical approach is able to identify a problem – that is, a clash of (a) personal beliefs with (b) a certain trade mark resulting in (c) negative emotions. However, because both clashing components in this scenario are mere matters of facts and both are detached from an objective moral framework, this begs the question why the trade mark must give way to get rid of the negative emotions caused by the clash.

Not only is an empirical approach unable to explain the superiority of personal beliefs or a “right not to be confronted with disturbing, abusive, insulting”^22^ marks, of which neither is self-evident but requires additional normative reasoning,^23^ especially since the ECtHR has consistently held that the ECHR “does not guarantee the right not to be confronted with opinions that are opposed to one’s own convictions”.^24^ In addition, one can ask whether it would be more sustainable to accept negative emotions in the short run, potentially making the public more “robust” against the trade mark in question and similar signs in the future, and potentially reducing emotional harm outside the area of trade mark law as well. This holds especially true since denying a sign trade mark protection pursuant to Art. 7(1)(f) EUTMR does not as such prevent the use of that sign in commerce or otherwise. Looking at empirical evidence,^25^ one could argue that morality bars are not (very) effective in reducing the use of such signs, in turn also rendering Art. 7(1)(f) EUTMR incapable of reducing harm by way of reducing the use of such signs.

#### Preventing the Perception of Endorsement

An empirical approach is also superfluous regarding the alleged aim of Art. 7(1)(f) EUTMR to prevent the perception of state endorsement of “immoral” subject matter.^26^ This is because an empirical assessment of the trade mark’s morality does not entail a normative judgment by the authorities in the sense that they assess compatibility with binding, objective values. Instead, it is a mere statement of facts about the subjective individual beliefs of a certain group of people. The moral judgment is delegated. In this framework, perceiving the act of registration as an act of endorsement is the result of a misunderstanding – the false assumption of a normative test where there is in fact only an empirical one. Employing an empirical test, however, does not solve that misunderstanding, only educating the public would do so.

#### Morality as a Subcategory of Distinctiveness

There seems to be only one rationale which could justify an empirical approach: treating morality as a subcategory of lack of distinctiveness. Some argue that signs contrary to morality are not capable of distinguishing, as the negative emotional impact caused by a conflict with the subjective values of the consumer encountering the sign superimposes the message regarding the origin of the goods. The message contrary to a person’s subjective values “will dominate source identification”.^27^ This understanding necessarily entails an empirical concept, since the assumption of superimposition builds on the actual effect the sign has on people.^28^ However, this assumption, and thus the idea that Art. 7(1)(f) EUTMR serves the primary goal of trade mark protection, is strongly contested.^29^ An understanding of Art. 7(1)(f) EUTMR as serving ancillary goals external to the main rationale of trade mark law is prevalent in the literature^30^ and inherent in the reasoning of both the Advocate General^31^ and the CJEU’s judgement, which is not at all concerned with distinctiveness. This is in line with the fact that acquired distinctiveness under Art. 7(3) EUTMR does not overcome Art. 7(1)(f) EUTMR.^32^

#### Conclusion: The Empirical Approach Is Unproportionate

Hence, an empirical approach makes Art. 7(1)(f) EUTMR ineffective in relation to the first three rationales analyzed above, meaning that these rationales are unable to justify the interference with freedom of speech, since a curtailment ineffective with regards to its aim cannot be proportionate. While an empirical approach would fit to the distinctiveness rationale, it seems implausible to assume that this rationale lies behind Art. 7(1)(f) EUTMR. Unless we can find another promising rationale,^33^ this means that an empirical approach to morality makes Art. 7(1)(f) EUTMR unproportionate – regardless of the other aspects that would otherwise have to be assessed in the proportionality test under Art. 10(2) ECHR, for example, the distinction between commercial expression and political or artistic expression and the specific margin of appreciation.^34^

### Opinion as the Basis to Restrict the Freedom of Expression Is Incompatible with the ECHR

A subjective empirical “bottom-up-approach” to morality is conceptionally essentially limited to a descriptive observation of public opinion at a specific point in time. Therefore, regardless of the foregoing observations, an empirical approach implies that what is or is not acceptable for the purpose of Art. 7(1)(f) EUTMR is defined by the opinion of (a portion of) the public (most likely the majority) at a certain point in time and thus serves to perpetuate and uphold this (majority) opinion. However, in a pluralistic society absolute conformity and consensus is impossible and also goes against the fundamental principles of pluralism, tolerance and broadmindedness inherent to every democratic society.^35^ These considerations relate back to the core essence of freedom of expression and the essential foundations of a democratic society.^36^ Likewise, they are a prerequisite for societal progress.^37^ Therefore, even expressions that may be received or regarded as offensive, shocking or disturbing are protected under the freedom of expression according to the settled case law of the ECtHR.^38^ Hence, there is a conflict between an empirical approach essentially focusing on (majority) opinion and the core concept of freedom of expression.^39^ Taking some portion of the population’s opinion as the benchmark for assessing morality under Art. 7(1)(f) EUTMR would favor the subjective beliefs of some portion of society over those of others. To borrow from Kennedy J: “A law that can be directed against speech found offensive to some portion of the public can be turned against minority and dissenting views to the detriment of all”.^40^

In an empirical understanding, the potential restriction of freedom of expression would be substantiated only with the subjective mores of the relevant public, independent of any (posited) objective values and irrespective of whether this empirical morality is in line with or even runs counter to objective values of morality.

When assessing whether measures to restrict advertisements were necessary under Art. 10(2) ECHR, the ECtHR has emphasized that merely relying on majority opinion to justify a restriction of freedom of speech “would be incompatible with the underlying values of the Convention if the exercise of Convention rights by a minority group were made conditional on its being accepted by the majority”.^41^ Otherwise “a minority group’s rights to, *inter alia*, freedom of expression would become merely theoretical rather than practical and effective as required by the Convention”.^42^ An empirical approach thus is unable to justify the curtailment of freedom of expression by Art. 7(1)(f) EUTMR, since it is not “necessary” in a democratic society in the sense of Art. 10(2) ECHR. Given that the ECtHR’s decision cited above concerned advertisements, this, again, holds true irrespective of the potentially commercial nature of the expression involved under Art. 7(1)(f) EUTMR.

### Conclusion: Against an Empirical Understanding of Morality

An empirical concept of “accepted principles of morality” would most likely mean that an interference with freedom of expression is *not* necessary in a democratic society and thus *un*justified. This makes a normative case against an interpretation of Art. 7(1)(f) EUTMR to include an empirical approach to morality.

## The CJEU’s Test for Morality: Empirical or Normative?

This result leads to the question: how empirical is the CJEU’s assessment under Art. 7(1)(f) EUTMR? It makes sense to conceptionally break the test down into two different components. First, how does the CJEU identify what the accepted principles of morality are (4.1)? Second, how does the court assess whether a mark is contrary to such principles (4.2)?^43^

### Accepted Principles of Morality as a Matter of Facts

According to the CJEU, the concept of accepted principles of morality “refers, in its usual sense, to the fundamental moral values and standards to which a society adheres at a given time.” These values and norms “are likely to change over time and vary in space” and “should be determined according to the social consensus prevailing in that society at the time of assessment”. Moreover, “due account is to be taken of the social context, including, where appropriate, the cultural, religious or philosophical diversities that characterise it, in order to assess objectively what that society considers to be morally acceptable at that time.”^44^

According to this understanding, accepted principles of morality on their face appear to be a matter of facts, that is, one of an actually existing social consensus (or shared beliefs) about what is morally acceptable at a given point in time and to a given portion of a given society, even though the CJEU does not explicitly describe this definition as relating to empirical questions.^45^ However, the fact that the AG explicitly considered accepted principles of morality to be of an empirical nature^46^ and the CJEU did not contest this statement in referring to the AG’s definition of morality also supports such an empirical understanding.

### Conflict of a Mark with Accepted Principles of Morality as a Mix of Matter of Facts and Normative Reasoning

The CJEU elaborated on the second component of the test, which in essence is about how the sign is perceived by the relevant public.^47^ The relevant public must not only find the sign to be in bad taste, but *perceive* it as contrary to accepted principles of morality as defined in the first component.^48^ This assessment “requires an examination of all the elements specific to the case”.^49^ Specifically, the CJEU states that the assessment must be

based on the perception of a reasonable person with average thresholds of sensitivity and tolerance, taking into account the context in which the mark may be encountered and, where appropriate, the particular circumstances of the part of the Union concerned. To that end, elements such as legislation and administrative practices, public opinion and, where appropriate, the way in which the relevant public has reacted in the past to that sign or similar signs, as well as any other factor which may make it possible to assess the perception of that public, are relevant.^50^

With notions like “the way in which the relevant public has reacted in the past” and “the context in which the mark may be encountered” this again appears as a matter of fact. However, the overall test sounds less factual/empirical than the first component and includes potentially normative elements. Interestingly enough, “public opinion” is only one of the elements to be taken into account.^51^

The most important point is the normative construction of a fictional person, on whose perception the whole assessment is to be based. The notion of “average thresholds of sensitivity and tolerance” ties in with the idea of some people being easily offended or not offended at all.^52^ It is potentially normative, because it filters out those opinions of members of the relevant public which are “extreme” *before* the empirical assessment takes place, not based on the specific mark in question, but as a general characteristic of a fictional member of the public. A pure factual assessment would include all members of the relevant public, giving less weight to the opinions of the extremes, but not completely disregarding them. Conveniently, factoring out the opinions of “extreme people” circumvents the hard question of quantitative thresholds by constructing a homogenous public from the very beginning. This is even more so regarding the notion of a “reasonable” person. While “average” could still be construed as being a matter of fact, what is “reasonable” is hardly an empirical question. This is, in the end, up to the deciding authorities. In sum, even if the relevant public does in fact perceive a sign as contrary to fundamental moral values, the deciding bodies can disregard this result by finding such perception unreasonable. It must be reiterated that this normative element only concerns the second component of the test, i.e. whether a sign is contrary to morality. The first component, defining the values making up the accepted principles of morality, still seems to be purely empirical.

## Potential Shortcomings of the CJEU’s Test

In light of freedom of expression, the CJEU’s test appears problematic for two reasons. First, insofar as the test is empirical, any refusal of “immoral” signs is tainted with the problems discussed in the Sect. 3 above. Second, the test seems to lack foreseeability. This puts pressure on the requirement that an interference with freedom of expression is “prescribed by law” under Art. 10(2) ECHR. Even though the broad wording of a provision restricting freedom of expression, such as the term “morality”, is not in and of itself problematic, it is necessary that its interpretation in practice is accessible to the affected party and foreseeable with regard to its effects, so that they are able to adjust their behavior accordingly.^53^ Consistent, clear and abundant case law can suffice to fulfill this requirement.^54^ Otherwise, the restriction imposed on freedom of expression may not be regarded as “prescribed by law” in the sense of Art. 10(2) ECHR, and thus not justified.^55^ It is doubtful that this judgment can be seen as a step towards the necessary clarification, especially given the de facto absence of traceable reasoning. The only interpretative tool used by the CJEU was that of a term’s usual meaning and the context in which it is generally used.^56^ In contrast to other decisions, however, the usual meaning of the term was not even established in relation to everyday language let alone the legislative context or the purpose of the provision.^57^ The only trace is a reference to the similar definition by the AG.^58^ This lack of any interpretive signposts takes away from the transparency of the decision and adds to the legal uncertainty surrounding the test. The lack of purposive interpretation also means that the judgment is of little help in answering the question whether Art. 7(1)(f) EUTMR does pursue legitimate aims (and is proportionate in relation to these aims) under Art. 10(2) ECHR.

## Conclusion

Although the CJEU mentioned freedom of expression, Art. 10 ECHR did not guide the interpretation of Art. 7(1)(f) EUTMR. As our analysis has shown, such guidance might have led away from an empirical approach to morality. The seemingly empirical elements of the CJEU’s test as well as its lack of clarity and foreseeability deserve a critical appraisal in light of freedom of expression. This could possibly be delivered indirectly, in a case concerning the morality bar of national trade mark law harmonised by Art. 4(1)(f) of the EU Trade Mark Directive, and potentially by the ECtHR itself. Given the numerous recent applications relating to the coronavirus across the EU, there should be no shortage of refusals based on morality in the near future.

## Footnotes

1 OJ 2017, L 154/1.

2 GC, Case T-69/17, *Fack Ju Göhte*, 24 January 2018, para. 29.

3 *See* AG, Case C-240/18 P, *Fack Ju Göhte*, 2 July 2019, paras. 45–57. The AG was also open to a reading of the GC’s statement under which freedom of expression potentially carries less weight in the balancing of interests in trade mark law compared to the other areas mentioned, *ibid*. para. 56.

4 CJEU, Case C-240/18 P, *Fack Ju Göhte*, 27 February 2020, para. 56. For the headnotes to this decision, see this issue of IIC at 10.1007/s40319-020-00937-8.

5 *See e.g*. CJEU, Case C-112/00, *Schmidberger*, 12 June 2003, para. 73; Case C-299/95, *Kremzow*, 29 May 1997, para. 14; Case C-260/89, *ERT*, 18 June 1991, para. 41; also *cf*. Art. 51(1) of the Charter of Fundamental Rights of the EU.

6 Recital 21 of EUTMR expressively states that it should be applied in a way that ensures full respect for fundamental rights and freedoms.

7 AG, Case C-240/18 P, *Fack Ju Göhte*, 2 July 2019, para. 52.

8 AG, *ibid*. para. 54.

9 The GC and EUIPO have regularly stated that Art. 7(1)(f) EUTMR is unable to breach freedom of expression (*see* OHIM, Case R-168/2011-1, *Fucking Freezing! by TÜRPITZ*, 1 September 2011, para. 15; GC, Case T-417/10, *HIJOPUTA*, 9 March 2012, para. 26; EUIPO (2020), Examination Guidelines, Part B, Section 4, Chapter 7, p. 3). This view is also shared by scholars (*see e.g.* Büscher (2020), para. 29; Hanf (2018), para. 132; Scassa (2013), p. 1191) and, regarding national trade mark law, by German courts (*see* Federal Patent Court, Case 26 W (pat) 69/05, *(Ehemaliges) DDR-Symbol der Sicherheitskräfte*, 17 July 2008, p. 13). However, both older and more recent decisions have discussed the interference of Art. 7(1)(f) EUTMR with freedom of expression (notably OHIM, Case R 495/2005-G, *SCREW YOU*, 6 July 2006, paras. 15–17, 24; EUIPO, Case R 2244/2016-2, *BREXIT*, 28 June 2017, paras. 25–35; Case R 875/2015-5, *HexaBody*, 7 March 2016, para. 13; Case R 2889/2014-4, *Die Wanderhure*, 28 May 2015, para. 12). So have national deciding bodies and scholarly literature (*see* Fhima (2013), pp. 298–302; Geiger and Pontes (2017); Hasselblatt (2018), para. 145), marking a trend towards accepting “that any refusal to register a mark on public policy/morality grounds constitutes an interference with the applicant’s right to freedom of expression […]” (Griffiths (2015), p. 427; *see also* Randazza (2016), p. 123).

10 ECtHR, Appl. No. 55153/12, *Dor v. Romania*, 25 August 2015, para. 44.

11 For an example of a seemingly empirical approach in practice *see*, *ex multis*, EUIPO, Case R 2244/2016-2, *BREXIT*, 28 June 2017, para. 18: “To be immoral a trade mark must be perceived by a substantial part of the relevant public […] as directly contrary to the accepted principles of morality in the context of current attitudes which prevailed at the filing date of the contested mark […]”.

12 *Cf.* AG, Case C-240/18 P, *Fack Ju Göhte*, 2 July 2019, paras. 77, 78.

13 *Cf.* AG, *ibid*. para. 79.

14 Due to Art. 52(3) CFR, the same applies to an interference with Art. 11 CFR, *cf*. Explanations Relating to the Charter of Fundamental Rights, OJ 2007, C 303/21 and C 303/33; CJEU, Case C-157/14, *Neptune*, 17 December 2015, para. 65; EUIPO, Case R 2244/2016-2, *BREXIT*, 28 June 2017, paras. 27, 28.

15 *See e.g.* ECtHR, Appl. No. 69317/14, *Sekmadienis Ltd. v. Lithuania*, 30 April 2018, para. 72; Geiger and Pontes (2017), p. 19; Griffiths (2015), p. 450.

16 Regarding the refusal of deceptive marks, the protection of the public against fraudulent commercial practices and respect for consumer law was found to be a legitimate aim, ECtHR, Appl. No. 55153/12, *Dor v. Romania*, 25 August 2015, para. 47.

17 *See e.g.* ECtHR, Appl. No. 55153/12, *Dor v. Romania*, 25 August 2015, para. 51. This is settled case law (ECtHR, Appl. No. 69317/14, *Sekmadienis Ltd. v. Lithuania*, 30 April 2018, para. 73; Appl. No. 16608/09, *Jakubowski v. Germany*, 23 June 1994, para. 26; Appl. No. 15450/89, *Casado Coca v. Spain*, 24 February 1994, para. 50; Appl. No. 10572/83, *Markt Intern Verlag GmbH und Klaus Beermann v. Germany*, 20 November 1989, para. 33) and reflected in the CJEU’s case law (CJEU, Case C-421/07, *Damgaard*, 2 April 2009, para. 27; Case C-380/03, *Tobacco Advertising II*, 12 November 2006, para. 155; Case C-71/02, *Karner*, 25 March 2004, para. 51; Case C-245/01, *RTL Television*, 23 October 2003, para. 73).

18 This selection limits our argument, since it potentially misses other rationales. However, it is worth pointing out that not every theory framed as “purpose” of Art. 7(1)(f) EUTMR is helpful for the analysis. For example, the notion that “immoral” trade marks do not deserve support by means of exclusive rights merely describes the effect of Art. 7(1)(f) EUTMR, i.e. preventing exclusive trade mark rights, as any other ground of refusal does. It does not, however, say anything about why that is the case (*cf*. *e.g.* EUIPO, Case R 803/2016-1, *La Mafia SE SIENTA A LA MESA*, 27 October 2016, para. 17; OHIM, Case R 495/2005-G, *SCREW YOU*, 6 July 2006, para. 13, arguing that government/administration should not positively assist the usage of offensive signs; also EUIPO, Case R 2244/2016-2, *BREXIT*, 28 June 2017, para. 12). The same goes for the idea that “immoral” marks do not justify spending of public resources (*see e.g.* Carpenter and Murphy (2011), p. 468, where it is further contested that this even correctly describes the effect of morality bars; Scassa (2013), p. 1187). For the argument that disputes about the registrability can be costlier than registration itself, *see e.g.* Farley (2015), p. 128; Gilson LaLonde and Gilson (2011), pp. 1482–1483; Schwender (2008), pp. 239–240. Others want to understand this “‘resources’ argument […] as another version of the argument against government support” which “should either factor into the endorsement analysis […] or be rejected as insufficient on its own” (*see* Tushnet (2016), p. 394).

19 This goal is often discussed with a view to so-called disparagement bars in trade mark law, *see e.g.* Grynbert (2016), p. 191; Farley (2015), p. 105; Scassa (2013), p. 1187; Greene (2008), p. 385.

20 The AG opined that the EUIPO does not have a “mandate” to introduce a “robust vision” of morality, “cut loose from (or, rather, much more stringent than) the one apparently prevailing in the Member State(s) in question”, AG, Case C-240/18 P, *Fack Ju Göhte*, 2 July 2019, para. 96. This is consistent with his proposal of an essentially empirical test (*see* AG, *ibid*. para. 80).

21 *See e.g.* Ziemer et al. (2017), p. 234; Farley (2014), p. 1050; Gilson LaLonde and Gilson (2011), p. 1485. For possible negative effects of such emotions on market efficiency *see e.g.* Snow (2017), pp. 1099–1100; Tushnet (2016), p. 388; also *cf*. GC, Case T-1/17, *La Mafia SE SIENTA A LA MESA*, 15 March 2018, para. 25, pointing in this direction by using the term “offense”, as well as OHIM, Case R 495/2005-G, *SCREW YOU*, 6 July 2006, para. 14.

22 Bonadio (2015), p. 61, and OHIM, Case R 495/2005-G, *SCREW YOU*, 6 July 2006, para. 14.

23 For an example of how to combine subjective offensiveness with objective values *see* ECtHR, Appl. No. 69317/14, *Sekmadienis Ltd. v. Lithuania*, 30 April 2018, para. 74, stating that “[…] the exercise of the freedom of expression carries with it duties and responsibilities. Amongst them, in the context of religious beliefs, is the general requirement to ensure the peaceful enjoyment of the rights guaranteed under Article 9 to the holders of such beliefs including a duty to avoid as far as possible an expression that is, in regard to objects of veneration, gratuitously offensive to others and profane”, however adding that “in a pluralist democratic society those who choose to exercise the freedom to manifest their religion cannot reasonably expect to be exempt from all criticism. They must tolerate and accept the denial by others of their religious beliefs and even the propagation by others of doctrines hostile to their faith”, ECtHR, *ibid*. para. 81.

24 ECtHR, Appl. No. 67667/09 and two others, *Bayev and others v. Russia*, 20 June 2017, para. 81.

25 *See* Carpenter and Garner (2015), pp. 362–364.

26 For this rationale *see e.g.* Ziemer et al. (2017), p. 233; Tushnet (2016), pp. 389–393; Ramsey (2016), p. 807; Gilson LaLonde and Gilson (2011), p. 1484; Abdel-Khalik (2007), pp. 211–212; Phillips and Simon (2004), p. 328; Baird (1993), p. 674. This rationale is disputed in German trade mark law, *see* German Federal Supreme Court, Case I ZB 89/11, *READY TO FUCK*, 2 October 2012, para. 18; Albrecht (2020), para. 653.

27 Grynbert (2016), p. 189.

28 There is an argument to be made that the idea of superimposing meanings goes well together with the obligations from the Paris Convention, because it includes an assessment of all factual circumstances, including the length of use, as required by Art. 6^quinquies^ C PC also in the case of telle-quelle marks which are rejected on grounds of morality according to Art. 6^quinquies^ B Nr. 3 PC, *see* Endrich-Laimböck (2019), p. 1034.

29 *See* Schwender (2008), p. 239; Abdel-Khalik (2007), p. 176; Baird (1993), p. 673.

30 *See* Endrich-Laimböck (2019), p. 1031.

31 *See* AG, Case C-240/18 P, *Fack Ju Göhte*, 2 July 2019, para. 57.

32 *See* CJEU, Case C-240/18 P, *Fack Ju Göhte*, 27 February 2020, para. 70. Moreover, the discussion of freedom of expression would otherwise shift towards questions more fundamental in nature, that is how the core subject matter requirement of distinctiveness and thus trade mark law as such go together with fundamental rights (*see* Endrich-Laimböck (2019), p. 1030). Presuming that trade mark law as such is compatible with freedom of expression, there would be no need to additionally justify Art. 7(1)(f) EUTMR.

33 *See supra* note 18.

34 *Cf.* Geiger and Pontes (2017), p. 27; *see also* ECtHR, Appl. No. 16632/12, *Csibi v. Romania*, 4 June 2019, paras. 42, 43, where the assessment regarding the refusal of a clearly political statement was assessed only in relation to commercial (not political) activities.

35 *Cf. e.g.* ECtHR, Appl. No. 69317/14, *Sekmadienis Ltd. v. Lithuania*, 30 April 2018, para. 70; Appl. No. 5493/72, *Handyside v. United Kingdom*, 7 December 1976, para. 49.

36 *Cf. e.g.* ECtHR, Appl. No. 69317/14, *Sekmadienis Ltd. v. Lithuania*, 30 April 2018, para. 70; Appl. No. 5493/72, *Handyside v. United Kingdom*, 7 December 1976, para. 49. These values are therefore also explicitly acknowledged to be part of the values on which the European Union is founded according to Art. 2 TEU (also *cf*. CJEU, Cases C-203/15 and C-698/15, *Tele 2 Sverige*, 21 December 2016, para. 93; EUIPO, Case R 2244/2016-2, *BREXIT*, 28 June 2017, para. 29).

37 *Cf.* ECtHR, Appl. No. 69317/14, *Sekmadienis Ltd. v. Lithuania*, 30 April 2018, para. 70; Appl. No. 5493/72, *Handyside v. United Kingdom*, 7 December 1976, para. 49. The ECtHR also underlined on that note that “any measures interfering with the freedom of assembly and expression other than in cases of incitement to violence or rejection of democratic principles – however shocking and unacceptable certain views or words used may appear to the authorities – do a disservice to democracy and often even endanger it. In a democratic society based on the rule of law, *the* ideas which challenge the existing order must be afforded a proper opportunity of expression”, *see* Appl. No. 10877/04, *Sergey Kuznetsov v. Russia*, 23 January 2009, para. 45.

38 *Cf. e.g.* ECtHR, Appl. No. 69317/14, *Sekmadienis Ltd. v. Lithuania*, 30 April 2018, para. 70; Appl. No. 5493/72, *Handyside v. United Kingdom*, 7 December 1976, para. 49.

39 *See also* EUIPO, Case R 2244/2016-2, *BREXIT*, 28 June 2017, para. 33.

40 *Matal v. Tam*, 137 S. Ct. 1744 [48 IIC 749 (2017)], opinion of Kennedy J (slip op.), p. 8 (available at https://www.supremecourt.gov/opinions/16pdf/15-1293_1o13.pdf).

41 ECtHR, Appl. No. 69317/14, *Sekmadienis Ltd. v. Lithuania*, 30 April 2018, para. 82; previously the ECtHR has additionally emphasised on that note that, even though “individual interests must on occasion be subordinated to those of a group, democracy does not simply mean that the views of the majority must always prevail: a balance must be achieved which ensures the fair and proper treatment of minorities and avoids any abuse of a dominant position”, *see e.g.* Appl. Nos. 4916/07, 25,924/08, 14,599/09, *Alekseyev v. Russia*, 11 April 2011, paras. 63, 81; Appl. No. 10519/03, *Barankevich v. Russia*, 26 July 2007, paras. 30–31.

42 ECtHR, Appl. No. 69317/14, *Sekmadienis Ltd. v. Lithuania*, 30 April 2018, para. 82.

43 This is not to say that the CJEU introduced some sort of two-step test, i.e. that these two components are applied subsequently. When applying Art. 7(1)(f) EUTMR to the case at hand, the CJEU did not even ask the first question and straightforward addressed the second component, i.e. whether the relevant public would perceive the sign as going against the fundamental moral values and standards of society, *see* CJEU, Case C-240/18 P, *Fack Ju Göhte*, 27 February 2020, paras. 69, 70.

44 CJEU, *ibid*. para. 39.

45 Especially the CJEU’s aim to “assess *objectively*” what society considers to be morally acceptable, could be understood to point either to an empirical or normative assessment.

46 AG, Case C-240/18 P, *Fack Ju Göhte*, 2 July 2019, para. 80.

47 CJEU, Case C-240/18 P, *Fack Ju Göhte*, 27 February 2020, para. 40.

48 CJEU, *ibid*. para. 41.

49 CJEU, *ibid*. para. 40.

50 CJEU, *ibid*. para. 42.

51 It is to be supplemented by *legislation and administrative practices*. The relevance of the latter raises the question whether legislation/administrative practice shall be treated as a mere indication of the underlying factual perception by the relevant public, or whether this means looking for objective values posited in the law or discernable from practice.

52 *Cf.* GC, Case T-69/17, *Fack Ju Göhte*, 24 January 2018, para. 34.

53 *Cf. e.g.* ECtHR, Appl. No. 69317/14, *Sekmadienis Ltd. v. Lithuania*, 30 April 2018, paras. 63, 64; Appl. No. 14622/89, *Hempfing v. Germany*, 7 March 1991.

54 *Cf.* ECtHR, Appl. No. 10572/83, *Markt Intern Verlag GmbH and Klaus Beermann v. Germany*, 20 November 1989, para. 30, regarding former Sec. 1 of the German Act against Unfair Competition (UWG), which also used to refer to “morality”.

55 *Cf.* ECtHR, Appl. No. 14622/89, *Hempfing v. Germany*, 7 March 1991. While the ECtHR found that the Romanian provision excluding deceptive marks passed the test and the refusal at hand was thus “prescribed by law” according to Art. 10(2) ECHR, it did not get a chance address the provision equivalent to Art. 7(1)(f) EUTMR, because the national courts had limited the refusal to deceptiveness, Appl. No. 55153/12, *Dor v. Romania*, 25 August 2015, paras. 45, 46.

56 And this only in relation to the first component of the test, i.e. the definition of accepted principles of morality, *see* CJEU, Case C-240/18 P, *Fack Ju Göhte*, 27 February 2020, para. 39.

57 *See* CJEU, Case C-476/17, *Pelham*, 29 July 2019, para. 28; Case C-516/17, *Spiegel Online*, 29 July 2019, para. 77.

58 *See* AG, Case C-240/18 P, *Fack Ju Göhte*, 2 July 2019, para. 77. The AG did not imply the usual-meaning argument himself. Also, it is worth pointing out that his considerations were not built on an isolated reading of the term, but on a conceptual distinction between “public policy” and “accepted principles of morality”. However, even if the first entails a top-down approach, it does not necessarily follow that the second must entail a bottom-up approach. There might be other dividing lines. For example, while both grounds could still be conceptualised as referring to objectively binding values, the first could relate to those objective values that can be discerned from the legal framework, while morality could relate to an unwritten normative order and its objectively binding societal values outside of positive law. The AG’s approach runs danger of a false dilemma fallacy, even if both terms must be distinguished. The need to distinguish is not self-evident, and a systematic approach is less persuasive than in relation to Sec. 2(a) of the Lanham Act, *see* Endrich-Laimböck (2019), p. 1032.
